# Effects of NatureKnit™ organic, a blend of organic fruit and vegetable fibers rich in naturally occurring bound polyphenols, on the metabolic activity and community composition of the human gut microbiome using the M-SHIME^®^ gastrointestinal model

**DOI:** 10.3389/fnut.2025.1740906

**Published:** 2026-01-16

**Authors:** Marlies Govaert, Cindy Duysburgh, Brendan Kesler, Massimo Marzorati

**Affiliations:** 1 ProDigest, Ghent, Belgium; 2 Van Drunen Farms and Futureceuticals, Momence, IL, United States; 3 Center for Microbial Ecology and Technology (CMET), Faculty of Bioscience Engineering, University of Ghent, Ghent, Belgium

**Keywords:** bound-polyphenols, gut microbiome, inulin, M-SHIME^®^, organic fiber, prebiotic, psyllium, short-chain fatty acid (SCFA)

## Abstract

**Objectives:**

The effects of a proprietary blend of organic fruit and vegetable fibers rich in naturally occurring bound polyphenols (commercially known as NatureKnit™ Organic) on the human gut microbiome were assessed.

**Methods:**

Short-term (48 h) *in vitro* colonic simulations using the validated Mucosal Simulator of the Human Intestinal Microbial Ecosystem (M-SHIME^®^) platform, with fecal inoculum from nine individual healthy human donors, were performed. Purified organic fibers (inulin and psyllium) were evaluated as comparators and a negative control was included. Primary measures included pH, gas pressure, short-chain fatty acid (SCFA) production, and microbial community composition.

**Results:**

All test products were well fermented with NatureKnit™ Organic showing slower fermentation kinetics than the purified fibers. SCFAs were significantly increased with all test products versus the negative control (*p* < 0.0001 for all) and NatureKnit™ Organic reached significance versus both purified fibers (*p* < 0.0001 for both). While relative abundances in the mucosal compartment were similar among all test conditions, luminal bacterial abundance increased with NatureKnit™ Organic and psyllium versus the negative control. The latter was mainly associated with statistically increased abundance (*p* < 0.05) of the genera *Eisenbergiella* and *Monoglobus*, with an additional strong enrichment of *Bacteroidaceae*. Furthermore, bacterial species richness was significantly increased with NatureKnit™ Organic versus the negative control (*p* = 0.0495), which was not observed for the purified organic fibers (*p* = 0.0567 and *p* = 0.4285 for inulin and psyllium, respectively).

**Conclusion:**

Overall, the obtained results indicate that NatureKnit™ Organic may have a greater and gentler prebiotic effect compared with established purified prebiotic fibers.

## 1 Introduction

In the U.S., over 90% of women and 97% of men do not meet the U.S. Adequate Intake level for fiber (1). This is primarily explained by the fact that over 85% of U.S. adults under-consume fruits, vegetables, and whole grains. Beyond North America, the fiber gap is also consistently considered a significant health issue, with fiber intake reported as insufficient across other continents as well (1–3). While a fiber intake of > 25 g/d is recommended, the average reported intake across continents only reaches 19.6 g/d, with several countries even remaining strongly below this level (1). Adequate fiber intake is an important part of a healthy dietary pattern, as the daily intake recommendations are based on levels that have been observed to reduce disease risk. Studies have shown that dietary fiber intake is negatively correlated with coronary heart disease, obesity, non-alcoholic fatty liver disease risk (4) and the prevalence of diabetic kidney disease among patients with type 2 diabetes (5). Further, total fiber, soluble fiber, and insoluble fiber intakes were associated with a lower risk of all-cause, cancer, and cardiovascular mortality among U.S. adults (6). Closing the gap between actual and recommended fiber intakes is an important public health goal.

In addition to being rich in fiber, plants such as fruits and vegetables are also rich in polyphenols. These phytochemical compounds provide health benefits, as a high dietary intake of polyphenols (such as in the Mediterranean diet) is associated with a reduction in the risk of numerous diseases, including cancer, cardiovascular disease, and neurodegenerative disorders (7). Further, polyphenols have been associated with beneficial effects in both preclinical and clinical studies of chronic diseases, including diabetes and obesity (8). Emergent research also shows that dietary polyphenols often occur in nature in association with dietary fiber. Based on the degree to which polyphenols are bonded to a food matrix, they may be classified as extractable (polyphenols which can be separated from food using aqueous or organic solvents) or non-extractable (polyphenols which cannot be separated from food using aqueous or organic solvents). These unique polyphenol classes also are metabolized differently within the human body with extractable polyphenols being primarily absorbed in the small intestine, while non-extractable polyphenols are not typically absorbed until they reach the large intestine where they are catabolized by colonic microbiota during fiber fermentation (9–12). Polyphenol metabolites produced during fermentation by colonic microbes have antioxidant activity and the ability to promote colon homeostasis and a balanced gut microbiota (9, 10, 13, 14). The vast gap in consumption of dietary fiber therefore may also lead to a reduction in consumption of fiber-bound-polyphenols that are capable of reaching and interacting with the gut microbiome.

Prebiotics consist of fibers and polyphenols that travel undigested through the gastrointestinal tract until they reach the colon, where they are partially or fully fermented by colonic bacteria (in a process called saccharolytic fermentation) resulting in a health benefit for the host that arises from alteration of the composition and/or activity of the gut microbiome (15, 16). Key metabolic byproducts of saccharolytic fermentation are short-chain fatty acids (SCFAs), which most commonly include acetate, propionate, and butyrate, known to be beneficial for human health (15, 17). In addition to promoting SCFA production, prebiotic fibers often support an enrichment of saccharolytic bacteria, such as *Bifidobacterium* and *Lactobacillus*, which can produce SCFAs (18, 19). Such shifts in the composition of the gut microbiota may lead to a decrease in the abundance of proteolytic bacteria and a subsequent reduction in branched chain fatty acids (BCFA) and other toxic byproducts of proteolytic fermentation. Several prebiotic classes (e.g., inulin) have been shown to be easily fermented by the gut microbiota, resulting in a predominantly proximal effect in the colonic region (20). Prebiotic compounds with more complex structures (e.g., arabinoxylans, polyphenols) may reach the distal colon (20), which is of particular interest as many colonic diseases occur in this colonic area (21).

Naturally occurring prebiotics are available in the form of dietary fibers, found in food sources such as fruits and vegetables (22). In fact, much of the available knowledge on the health benefits of fiber is derived from studies that evaluated the effects of dietary fibers in food rather than commercially available prebiotic fiber supplements (23). Considering that most people in the U.S. currently under-consume fiber, there is continued and growing interest in utilizing fiber supplements to help lessen the gap between fiber consumption from foods and fiber intake goals. For example, inulin and psyllium are two widely commercialized purified fibers that are proven prebiotics (24–29). It is important to note that purified fibers, which have typically been extracted out of their original food matrix, typically lack the minerals, vitamins, polyphenols, and other nutrients found in fiber-rich fruits and vegetables (22). On top of the absence of additional micronutrients, purified fibers may be rapidly fermented, potentially leading to bloating and gastrointestinal distress (30, 31).

Organically grown foods cannot be genetically modified and must be grown without the use of most synthetic fertilizers and pesticides. Organic foods have been gaining in popularity among consumers for a variety of reasons, including health and wellbeing, environmental concerns, and/or consideration of the impact on animal welfare (32), while there is also growing evidence that organic foods contains higher antioxidant levels (particularly polyphenols) as compared to conventional foods (32). Some short-term studies have demonstrated potential beneficial effects from organic diets, such as dramatically reduced levels of pesticide metabolite excretion in the urine following a switch to an organic diet (33, 34). This finding is of great interest, though the clinical implications are currently unknown. Observational studies have reported positive associations between an organic diet and several outcomes, including birth defects, allergic sensations, metabolic syndrome, and fertility (32). The nature of observational studies, however, limits the ability to determine the causality of these associations and additional research is critical to better understand the health effects of organic (versus conventional) foods. Further, the increased consumer interest in organic food products, regardless of well-established differentiation in health outcomes, warrants such research efforts.

In a previous study with a conventional, non-organic version of NatureKnit™, a blend of fruit and vegetable fibers rich in naturally occurring polyphenols, supplementation demonstrated prebiotic effects in an *in vitro* short-term colonic simulation model with three healthy fecal donors (35). This previous study reported significant stimulation in SCFA production accompanied with increased bacterial abundance and diversity versus well-established purified prebiotic fibers, thereby showing a slower fermentation process (35). In the current study, we evaluated NatureKnit™ Organic and two well-established organic prebiotic fibers, inulin and psyllium husk, for their fermentation characteristics and their effects on the metabolism and composition of the gut microbiota of nine healthy adult donors using the short-term *in vitro* Mucosal Simulator of the Human Intestinal Microbial Ecosystem (M-SHIME^®^) platform. This study expands upon the previous findings reported for non-organic NatureKnit™ and uniquely investigates the metabolism of organic prebiotics (35).

## 2 Materials and methods

### 2.1 Test products

NatureKnit™ Organic, a commercial plant-based fiber blend comprised of a proprietary mixture of organic apple, grape, and blueberry fibers, as well as organic whole spinach and carrot, was tested during the current study. A total of at least 40% dietary fiber and 1–4% of naturally occurring fiber-bound polyphenols was present in the blend, compounds which would reach the colon intact following intake (Futureceuticals, Momence, IL, United States). Organic versions of two common purified fibers, inulin and psyllium, were used as comparator products (specifications of the purified fibers are available upon request).

### 2.2 Short-term colonic simulation

Fresh human fecal samples were collected from nine healthy adults (female, *n* = 4; male, *n* = 5). The donors had a healthy body mass index (18.5 to 24.9), were not diagnosed with any diseases that could result in gut microbiome dysbiosis (e.g., diabetes, Parkinson’s disease, irritable bowel syndrome, inflammatory bowel disease), had not taken antibiotics during the 4 months before fecal sample collection, followed a Western diet pattern, and were aged between 24 and 38 years. Following sample collection, a fecal slurry was prepared from each sample by adding an anaerobic phosphate buffered saline buffer in an anaerobic atmosphere. Next, brief centrifugation was applied to remove the remaining large particles, followed by adding an equal volume of optimized in-house cryoprotectant [modified from Hoefman et al. (36)] and flash freezing. The collection and use of fecal materials was conducted as approved by the Ethics Committee of the University Hospital Ghent (reference number ONZ-2022-0267, approved on 29 July 2022). Informed consent was obtained from all subjects involved in the study.

A short-term single-stage colonic M-SHIME^®^ experiment was performed to assess microbial metabolic activity and community composition following the fermentation of the test products in this simulated colon model (37–39). Multiple studies have validated this model for its representativeness of the human *in vivo* situation (40–43) and it has been used extensively to test the impact of various test products/conditions on the human gut microbiota (44–48). At initiation, 63 mL fresh carbohydrate-depleted medium representative for the colonic environment (nutritional medium PD01; ProDigest, Ghent, Belgium) was added to each reactor, followed by the addition of either no test product (negative control) or one of the test products (NatureKnit™ Organic, organic inulin, organic psyllium; fiber-matched so that each test condition received 1.667 g fiber/L). The colonic *in vitro* dose of the test products corresponded to the target human dose of 2.5 g. Next, 7 mL of de-frosted cryopreserved fecal inoculum from one of the nine healthy donors was added to the appropriate reactors, resulting in nine biological replicates per test condition. Simulation of the mucosal layer was achieved by the addition of mucus-coated carriers, prepared as described in Van den Abbeele et al. (38), into each colonic reactor. To create an anaerobic environment, each of the reactors was flushed with nitrogen gas. Incubations were caried out for 48 h at 37 °C with continuous mild shaking (90 rpm). All individual donor-product conditions were performed in a single technical replicate.

At 0 h, 6 h, 24 h, and 48 h, pH and gas pressure were measured, and samples were collected to determine SCFA, lactate, BCFA, and ammonium concentrations. These data were used for fermentation profiling and assessment of microbial metabolic activity. Samples were collected at 0 h (negative control only), 24 h, and 48 h to determine the luminal microbial community composition and at 48 h to assess the mucosal microbial community composition.

### 2.3 Fermentation profiling and microbial metabolic activity

A Senseline F410 pH meter (ProSense, Oosterhout, Netherlands) was used to measure pH changes. Gas pressure was measured using a hand-held pressure indicator (CPH6200; Wika, Echt, Netherlands). SCFAs (acetate, propionate, and butyrate) and BCFAs were measured based on the methods of De Weirdt et al. (49). Briefly, SCFA were extracted from the samples with acetonitrile, following the addition of 2-methyl hexanoic acid as an internal standard. Extracts were analyzed using a GC-2030 gas chromatograph (Shimadzu, ‘s-Hertogenbosch, Netherlands), equipped with a capillary SH-PolarD column (dimensions: 30 mm x 0.32 mm x 1.00 μm; Shimadzu), a flame ionization detector and a split injector. The injection volume was 1 μL and the temperature profile was set from 110 to 160 °C with a temperature increase of 6 °C/min. The carrier gas was nitrogen and the temperature of the injector and detector were set at 200 °C. An Enzytec™ kit (R-Biopharm, Darmstadt, Germany) was used according to the manufacturer’s instructions for measuring lactate concentrations. Levels of ammonium (measured as ammonium-nitrogen [NH_4_^+^-N]) were quantified using the method of Tzollas et al. (50).

### 2.4 DNA extraction and 16S rRNA sequencing

The method described by Duysburgh et al. was used to isolate total DNA (51). Primers spanning two hypervariable regions (V3–V4) of the 16S rRNA gene, 341F, 5′-CCTACGGGNGGCWGCAG-3′ and 785R, 5′-GACTACHVGGGTATCTAAKCC-3′ were used for 16S-targeted sequencing (52, 53). PCR amplification and library construction were performed as reported by Kozich et al. (53). A pair-end sequencing approach was used using the Illumina by MiSeq platform and sequencing of 2 × 250 bp resulted in 424 bp amplicons (LGC Genomics GmbH, Berlin, Germany), which are taxonomically more informative than smaller fragments. Read assembly and cleanup was accomplished using the Schloss lab MiSeq protocol (53, 54). Briefly, reads were assembled into contigs, alignment-based quality filtering was performed (alignment to the mothur-reconstructed SILVA SEED alignment, v138), chimeras were removed (vsearch v2.13.3), taxonomy was assigned using a naïve Bayesian classifier (55) and SILVA NR v138_1, and contigs were clustered into Operational Taxonomic Units (OTUs) at 97% sequence similarity using mothur (v.1.44.3). Sequences that could not be classified or were classified as Eukaryota, Archaea, chloroplasts, or mitochondria were removed. Each OTU was represented by the most abundant sequence within that OTU.

### 2.5 Quantification of total bacterial cells in the luminal samples

The total number of bacterial cells in the luminal samples was quantified using flow cytometry (BD Accuri C6 Plus Flow Cytometer [BD Biosciences, Franklin Lakes, NJ, United States]; high flow rate setting). Bacterial cells were separated from signal noise and medium debris by applying a SYTO channel threshold of 700. Quantification of the bacterial cells enabled the conversion of the metagenomics data from relative abundances to absolute abundances. This was accomplished by multiplying relative abundances in a sample with the total cell count determined by flow cytometry (56).

### 2.6 Statistical analysis

Differences in pH, gas pressure, SCFA, lactate, BCFA, and ammonium were assessed between 0 h and 48 h incubation (i.e., over the entire incubation period). Statistically significant differences between different test conditions were determined using a repeated measures one-way ANOVA with post-hoc Benjamini-Hochberg tests (*p* < 0.05) using GraphPad Prism (v10.6.0).

Alpha diversity was analyzed using three common indices: the observed taxa index, which indicates species richness; the Shannon index, which indicates species richness and evenness; and the Inverse Simpson index, which indicates species richness and evenness, giving more weight to common or dominant species. Alpha diversity indices were calculated using phyloseq v1.44.0 (57). Repeated measures one-way ANOVA with post-hoc Benjamini-Hochberg tests were used to determine significant differences between the different test conditions in terms of genus richness or evenness (*p* < 0.05) using GraphPad Prism (v10.6.0).

Differential abundance analysis was performed using Linear Discriminant Effect Size (LEfSe) (58) and treeclimbR (59) to identify the taxa most likely to explain differences between treatments. LEfSe was performed on relative abundance data obtained by total-sum scaling. Linear Discriminant Analysis (LDA) scores between treatment and individual taxon abundances were calculated using MASS (v7.3.58-3). Significant features met *p* ≤ 0.05 for Kruskal-Wallis and Wilcoxon tests, with no minimal score restrictions for LDA, though a score of ≥2 is generally considered biologically relevant. With treeclimbR, bacterial enrichments were considered statistically significant if they had a −log(*p*-value) > 1.3. Four categories were used for taxa classification: (1) not significant and not biologically relevant (−2 < log_2_ fold change [FC] < +2, and −log_10_[*p*-value] < 1.3), (2) biologically relevant, but not statistically significant (log_2_FC < −2 or log_2_FC > +2, and −log_10_[*p*-value] < 1.3), (3) statistically significant, but not biologically relevant (−2 < log_2_FC < +2, and −log_10_[p-value] > 1.3), and (4) biologically and statistically significant (log_2_FC < −2 or log_2_FC > +2, and −log_10_[p-value] > 1.3). TreeclimbR analysis was run using treeclimbR v0.1.5 and edgeR v3.42.421. Benjamini-Hochberg multiple testing correction was used, and the alpha-level was set at 0.05.

## 3 Results

### 3.1 Fermentation profile

**Figure 1:**
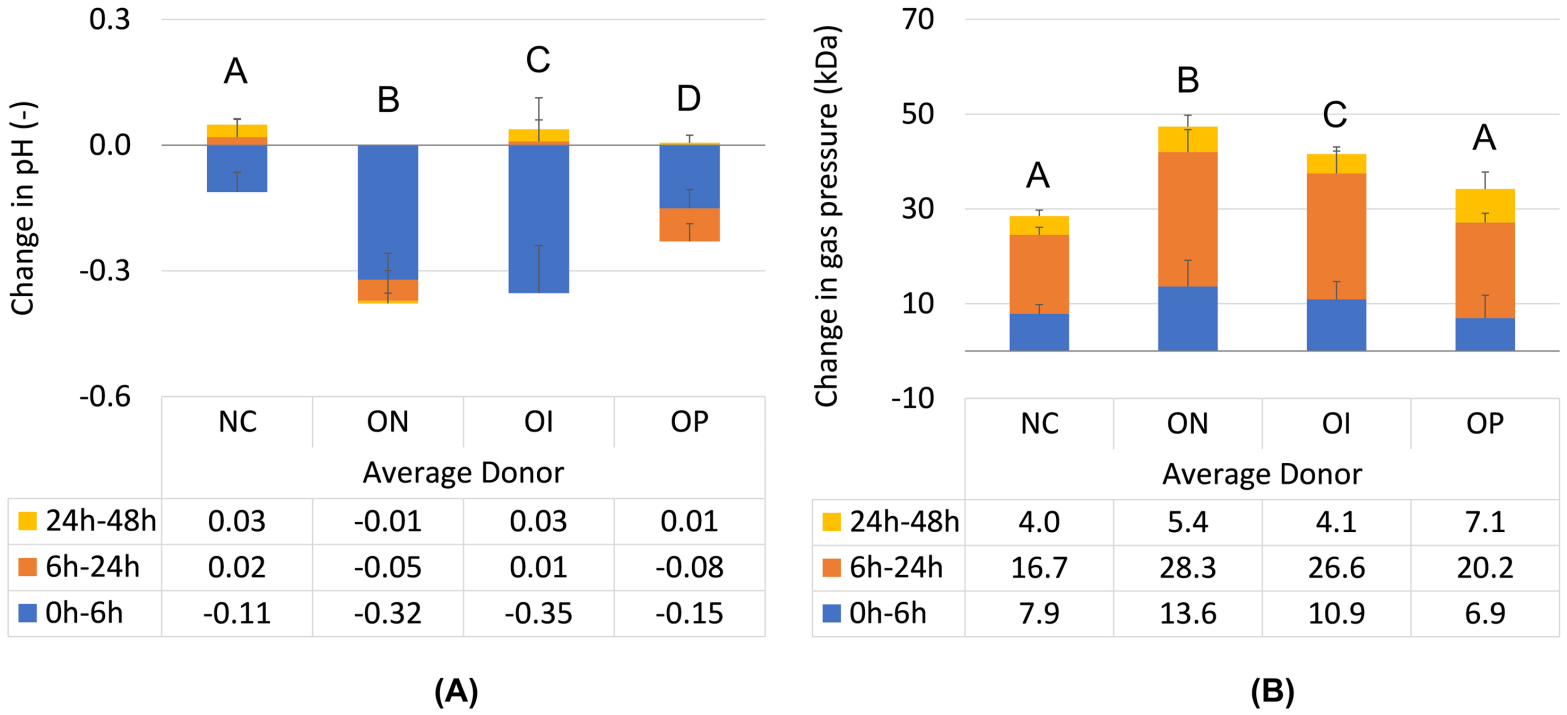
Changes in **(A)** pH (−) and **(B)** gas pressure (kPa) during different time intervals (0 h–6 h, 6 h–24 h, and 24 h–48 h) following test product administration in M-SHIME^®^ short-term colonic incubations. Incubations included the negative control (colonic incubation blank medium), NatureKnit™ Organic (1.667 g fiber/L), organic inulin (1.667 g fiber/L), and organic psyllium (1.667 g fiber/L). Results are presented as mean ± standard deviation across donors (*n* = 9). Statistical analysis was performed over the course of the entire colonic incubation phase (i.e., between 0 h and 48 h). Repeated measures one-way ANOVA with *post hoc* Benjamini-Hochberg tests were used to compare changes between the different test conditions. A *p*-value of <0.05 was considered statistically significant. When test conditions do not share a same letter statistically significant differences between these test conditions were observed, while no significant differences were observed between test conditions that share a same letter. M-SHIME^®^, Mucosal Simulator of the Human Intestinal Microbial Ecosystem; NC, negative control; OI, organic inulin; ON, Organic NatureKnit™; OP, organic psyllium.

Changes in pH and gas pressure across donors are shown in Figure 1. Across donors, the pH of the negative control reactors decreased during the first 6 h of incubation and remained relatively stable from 6 h to 24 h and from 24 h to 48 h (Figure 1A; Supplementary Table S1). All three test products had a significantly greater decrease in pH over the 48 h incubation compared with the negative control (*p* < 0.0001 for all). Supplementation with NatureKnit™ Organic had the largest overall pH decrease (reaching significance; *p* < 0.0001 both versus inulin and psyllium), followed by organic inulin (*p* = 0.0006 versus psyllium) and organic psyllium. NatureKnit™ Organic was the only test product that demonstrated a continuous decrease in pH over the entire incubation period. Supplementation with organic inulin resulted in an initial pH decrease followed by an increase at 6 h–24 h and 24 h–48 h. Supplementation with organic psyllium resulted in a decrease in pH at both the 0 h–6 h and 6 h–24 h timepoints, followed by an increase at the 24 h–48 h timepoint.

Gas pressure increased over time for the negative control and all three test products (Figure 1B). Gas pressure was significantly greater versus negative control for both NatureKnit™ Organic and organic inulin supplementation (*p* < 0.0001 for both), and not significantly different between the negative control and organic psyllium-supplemented reactors (*p* = 0.0544). Gas pressure was highest with NatureKnit™ Organic supplementation, reaching significance compared to the other organic test products (*p* = 0.0009 both versus inulin and psyllium), though remaining within normal physiological range (<100 kPa).

### 3.2 Microbial metabolic activity

**Figure 2:**
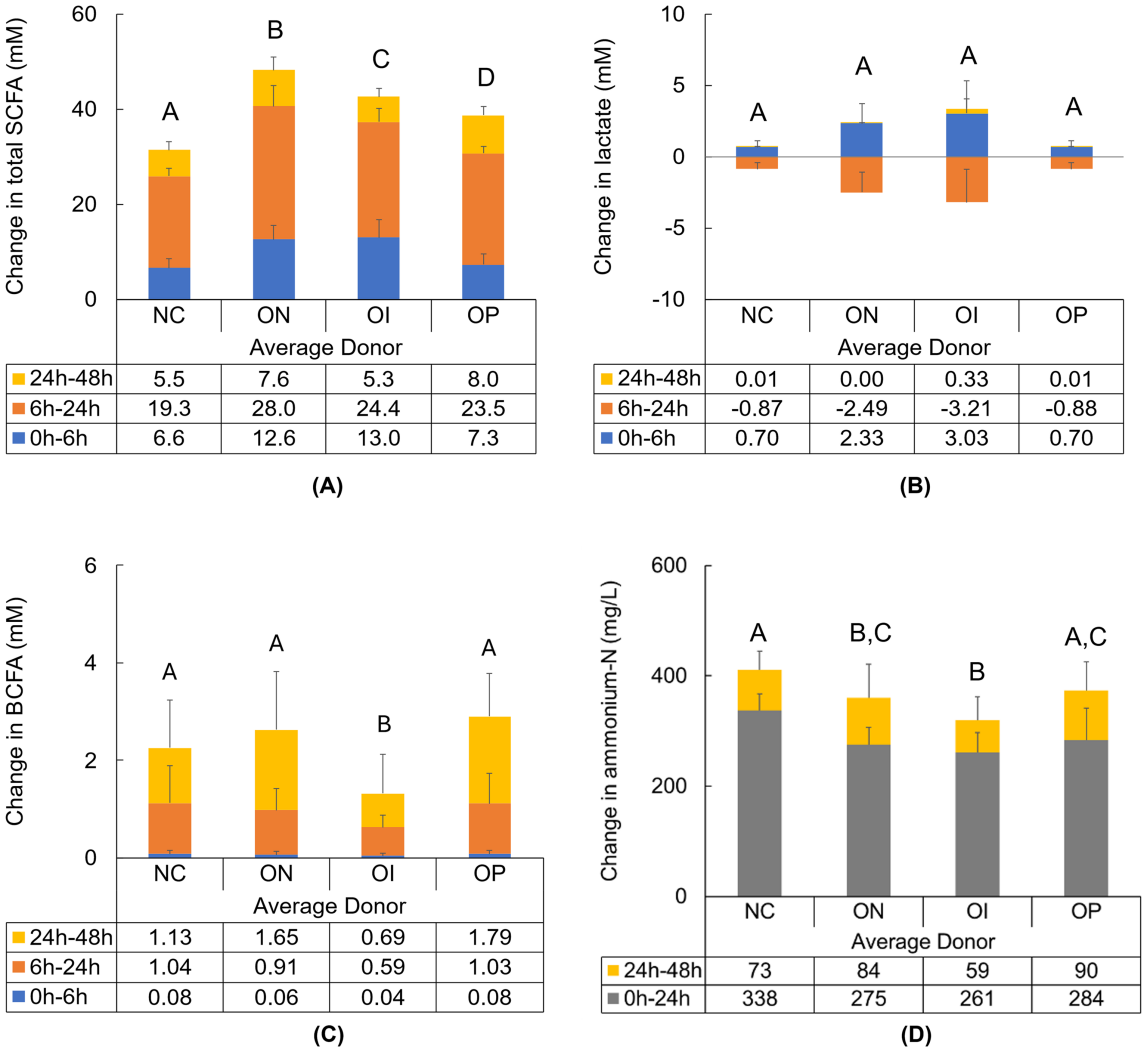
Changes in **(A)** total SCFA (mM), **(B)** lactate (mM), **(C)** BCFA (mM), and **(D)** ammonium-N (mg/L) during different time intervals (0 h–6 h, 6 h–24 h, and 24 h–48 h) following test product administration in M-SHIME^®^ short-term colonic incubations. Incubations included the negative control (colonic incubation blank medium), NatureKnit™ Organic (1.667 g fiber/L), organic inulin (1.667 g fiber/L), and organic psyllium (1.667 g fiber/L). Results are presented as mean ± standard deviation across donors (*n* = 9). Statistical analysis was performed over the course of the entire colonic incubation phase (i.e., between 0 h and 48 h). Repeated measures one-way ANOVA with post hoc Benjamini-Hochberg tests were used to compare changes between the different test conditions. A *p*-value of <0.05 was considered statistically significant. When test conditions do not share a same letter statistically significant differences between these test conditions were observed, while no significant differences were observed between test conditions that share a same letter. BCFA, branched chain fatty acid; M-SHIME^®^, Mucosal Simulator of the Human Intestinal Microbial Ecosystem; NC, negative control; OI, organic inulin; ON, Organic NatureKnit™; OP, organic psyllium; SCFA, short-chain fatty acid.

Changes in microbial metabolic activity across donors are shown in Figure 2. Changes in total SCFA levels were most pronounced over the 6 h–24 h timepoint (Figure 2A). Across donors, total SCFA levels significantly increased with each test product versus the negative control (*p* < 0.0001 for all), with the greatest overall increase observed with NatureKnit™ Organic supplementation (reaching significance; *p* < 0.0001 both versus inulin and psyllium; 0 h–6 h, +12.6 mM; 6 h–24 h, +28.0 mM; 24 h–48 h, +7.6 mM), followed by organic inulin (*p* = 0.0140 versus psyllium; 0 h–6 h, +13.0 mM; 6 h–24 h, +24.4 mM; 24 h–48 h, +5.3 mM) and organic psyllium (0 h–6 h, +7.3 mM; 6 h–24 h, +23.5 mM; 24 h–48 h, +8.0 mM). NatureKnit™ Organic also resulted in a significantly higher overall production of acetate (+28.9 mM; *p* = 0.0190 and *p* < 0.0001 versus inulin and psyllium, respectively), propionate (+12.2 mM; *p* < 0.0001 and *p* = 0.0171 versus inulin and psyllium, respectively), and butyrate (+4.3 mM; *p* = 0.0685 and *p* = 0.0037 versus inulin and psyllium, respectively), compared to organic inulin (acetate, +27.5 mM; propionate, +9.7 mM; butyrate, +4.0 mM) and organic psyllium (acetate, +21.8 mM; propionate, +10.4 mM; butyrate, +3.4 mM) (data not shown). For all test conditions, lactate levels increased at 0 h–6 h and decreased at 6 h–24 h (Figure 2B). At the 24 h–48 h timepoint, there was essentially no change in lactate with the negative control, Organic NatureKnit™, and organic psyllium, but there was a slight increase with organic inulin. The overall lactate levels were similar for all test conditions (*p* > 0.05 for all).

BCFA levels increased over time for all test conditions, including the negative control (Figure 2C). Very little increase was observed initially, with the main increases occurring in the 6 h–24 h and 24 h–48 h time periods. Compared with the negative control, overall BCFA levels were not significantly different with NatureKnit™ Organic (*p* = 0.4201) or organic psyllium (*p* = 0.2246), but were significantly lower with organic inulin (*p* = 0.0244). NH_4_^+^-N levels increased over time, with the greatest increases observed 0 h–24 h, and were lower across donors for all test products compared with the negative control, reaching significance for organic inulin (*p* = 0.0044) with a similar tendency for NatureKnit™ Organic (*p* = 0.0770) (Figure 2D).

### 3.3 Microbial community composition

#### 3.3.1 Luminal gut microbial abundance

**Figure 3:**
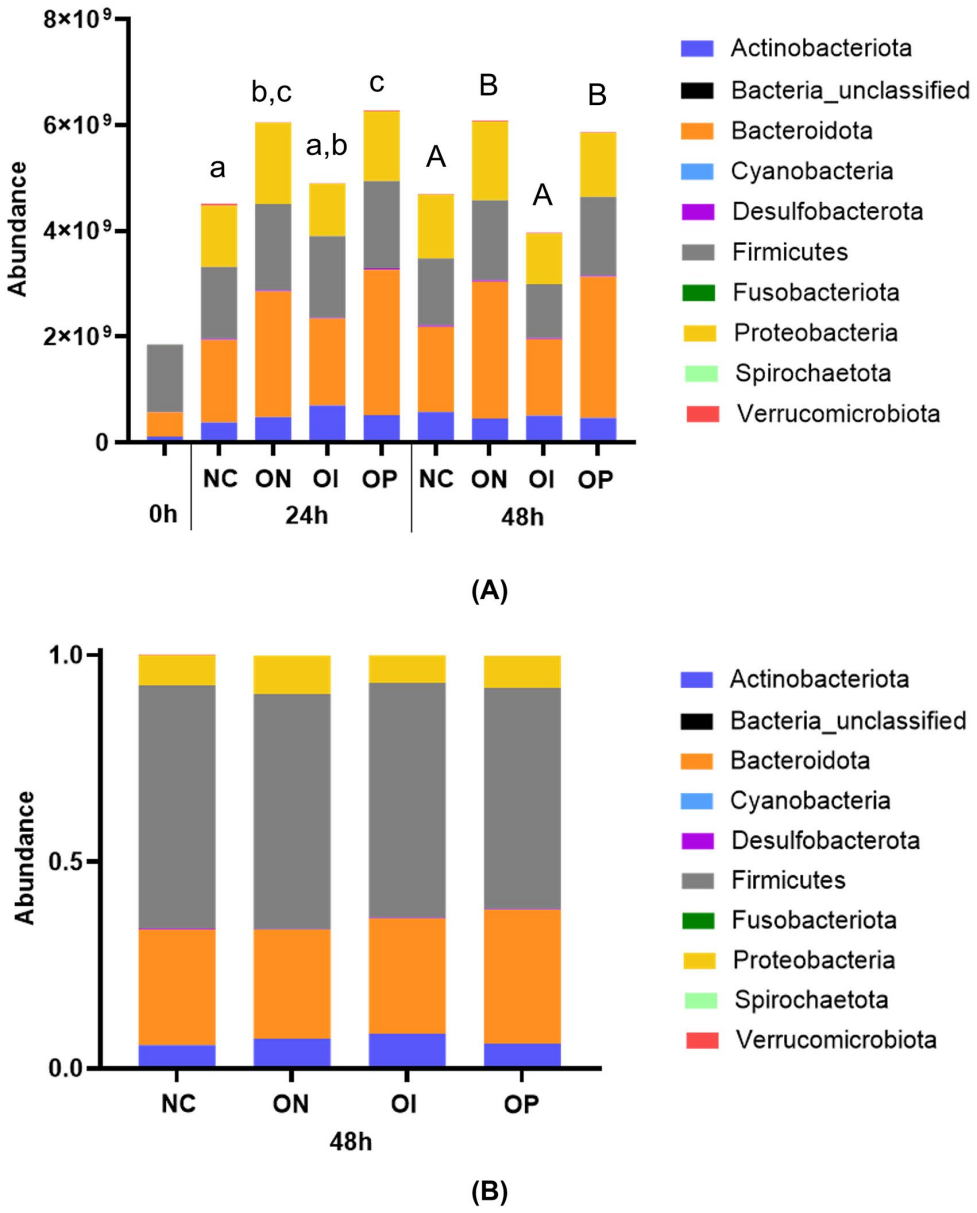
Stacked bar plots showing **(A)** absolute phyla abundances (cells/mL) in the lumen compartment and **(B)** relative phyla abundances (%) in the mucosal compartment. Incubations included the negative control (colonic incubation blank medium), NatureKnit™ Organic (1.667 g fiber/L), organic inulin (1.667 g fiber/L), and organic psyllium (1.667 g fiber/L). Results are presented as average across donors (*n* = 9). Flow cytometry was used to determine the total number of bacterial cells in the luminal samples. Repeated measures one-way ANOVA with post hoc Benjamini-Hochberg tests were used to compare changes in total bacterial abundance between the different test conditions. A *p*-value of <0.05 was considered statistically significant. When test conditions do not share a same letter statistically significant differences between these test conditions were observed, while no significant differences were observed between test conditions that share a same letter (small letters for the 24 h time point, capital letters for the 48 h time point). NC, negative control; OI, organic inulin; ON, Organic NatureKnit™; OP, organic psyllium.

The phylum-level bacterial community composition is shown in Figure 3. In the luminal compartment, the main phyla for all test conditions were Bacteroidota, Firmicutes, Proteobacteria, and Actinobacteriota (Figure 3A). Across donors, the absolute bacterial abundance tended to increase with all test products relative to the negative control after 24 h of colonic incubation (Organic NatureKnit™, +33.9%; organic inulin, +8.2%; organic psyllium, +38.8%), reaching significance for NatureKnit™ Organic (*p* = 0.0253) and organic psyllium (*p* = 0.0114). At the 48 h time point, only NatureKnit™ Organic (+29.8%; *p* = 0.0006) and organic psyllium (+25.0%; *p* = 0.0032) significantly increased total bacterial abundance versus the negative control, while a lower abundance was observed following inulin supplementation (−15.4%; *p* = 0.0605). Overall, highest bacterial abundance was observed following NatureKnit™ Organic supplementation, reaching significantly higher levels as compared to the negative control (*p* = 0.0006) and inulin (*p* < 0.0001), while similar bacterial abundance was observed for organic psyllium (*p* = 0.5564). In particular, levels of Bacteroidota increased in abundance at both 24 h and 48 h with NatureKnit™ Organic and organic psyllium supplementation compared with the negative control. Luminal data for individual donors is shown in Supplementary Figure S1.

**Figure 4:**
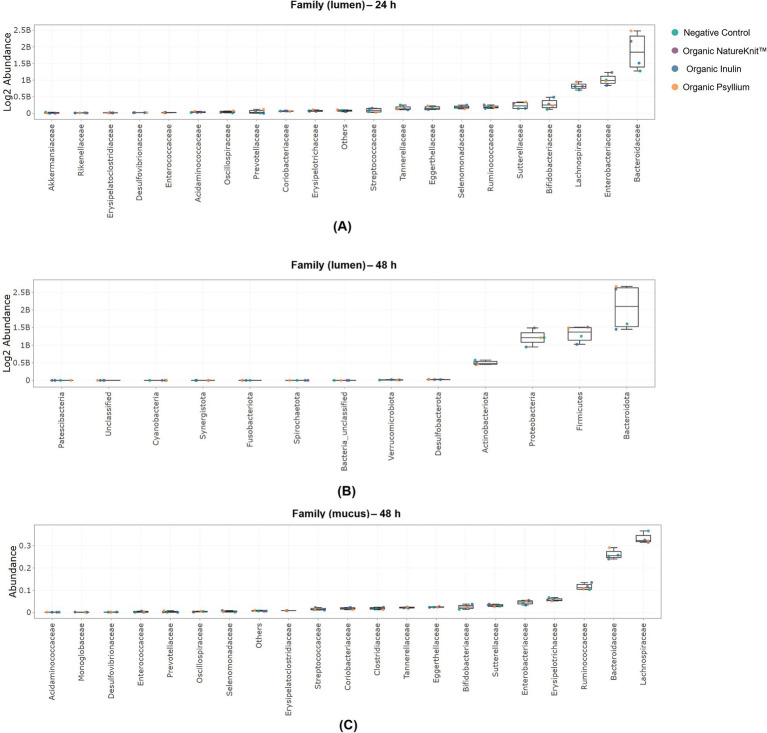
Jitter plots showing **(A)** the absolute abundance (cells/mL) of the top 20 most abundant families in the lumen compartment after 24 h, **(B)** the absolute abundance (cells/mL) of the top 20 most abundant families in the lumen compartment after 48 h, and **(C)** the relative abundance (−) of the top 20 most abundant families in the mucosal compartment after 48 h. Incubations included the negative control (colonic incubation blank medium), NatureKnit™ Organic (1.667 g fiber/L), organic inulin (1.667 g fiber/L), and organic psyllium (1.667 g fiber/L). Each dot represents the average across donors (*n* = 9).

Treatment effects on the absolute bacterial abundance in the luminal compartment are shown in Supplementary Figure S3, Figure 4, and Table 1. At 24 h in the lumen, an enrichment of Bacteroidota was observed with NatureKnit™ Organic and organic psyllium, but not with organic inulin (Supplementary Figure S3A). The Bacteroidota enrichment was linked to an enrichment of *Bacteroidaceae* at the family level (Figure 4A). For organic psyllium, this could be linked to a statistically and biologically significant enrichment (*p* < 0.05 and FC > 4) of the *Bacteroides* genus (Table 1). The Firmicutes phylum was increased for all treatment conditions compared to the negative control, mainly linked to an enrichment of the *Lachnospiraceae* family, with different genera being statistically enriched (*p* < 0.05) for the different test products. Proteobacteria levels were also enriched following product supplementation (except for organic inulin) compared with the negative control. This was mainly due to an increased abundance in the *Enterobacteriaceae* and/or *Sutterellaceae* family, though not resulting in statistical enrichments at genus level. The Actinobacteriota phylum was upregulated for all product-conditions, linked to an increased abundance in the *Bifidobacteriaceae* family, reaching statistical and biological significance (*p* < 0.05 and FC > 4) for the *Bifidobacterium* genus following supplementation with organic inulin. At 48 h, the results were largely similar to those at 24 h (Supplementary Figure S3B; Figure 4B). The specific increase in bacterial abundance for NatureKnit™ Organic and organic psyllium could be linked to consistent statistical enrichment (*p* < 0.05 and FC < 4) of the genera *Eisenbergiella* and *Monoglobus* (among other product-specific increases).

**Table 1:** Overview of treatment-induced bacterial enrichments (+) at genus level in the different colon environments (lumen and mucus) after 24 h and 48 h of colonic incubation, as identified with LEfSe and/or treeclimbR.

Phylum	Family	Genus	24 h Lumen			48 h Lumen			48 h Mucus		
			ON	OI	OP	ON	OI	OP	ON	OI	OP
Actinobacteriota	*Bifidobacteriaceae*	*Bifidobacterium*		+						+	
Bacteroidota	*Bacteroidaceae*	*Bacteroides*			+						
	*Tannerellaceae*	*Parabacteroides*		+				+			
Firmicutes	*Lachnospiraceae*	*Anaerostipes*				+					
		*Dorea*		+							
		*Eisenbergiella*			+	+		+			+
		*[Eubacterium] hallii group*					+				
		*Lachnospiraceae ND3007 group*			+	+					+
		*Lachnospiraceae NK4A136 group*				+					
		*F_Lachnospiraceae*		+							
	*Monoglobaceae*	*Monoglobus*	+		+	+		+			
	*Oscillospiraceae*	*F_Oscillospiraceae*		+							

Colors indicate the type of significance, with red color showing statistically and biologically significant enrichments (*p* < 0.05 and FC > 4), blue color showing statistically significant enrichments (*p* < 0.05 and FC < 4) and grey color showing no significant enrichments (*p* > 0.05 and FC < 4).

#### 3.3.2 Mucosal gut microbial abundance

Within the mucosal environment, relative abundances on the phylum level were similar as within the lumen among the different test conditions, including the negative control, across all donors (Figure 3B). The main phyla were Firmicutes, Bacteroidota, Proteobacteria, and Actinobacteriota. Mucosal data for individual donors is shown in Supplementary Figure S2. Overall, individual data showed a more consistent effect across donors following supplementation with NatureKnit™ Organic as compared to treatment with organic inulin and organic psyllium.

Treatment effects on the relative bacterial abundance in the mucosal compartment is shown in Supplementary Figure S3C, Figure 4C, and Table 1. In the mucosal compartment (48 h), the Firmicutes phylum was reduced by all test products compared with the negative control, mainly associated with reduced abundance of the highly abundant *Lachnospiraceae* family, while *Ruminococcaceae* abundance increased following product supplementation, though not reaching statistical significance at genus level. Mucosal Bacteroidota abundance, on the other hand, increased for most test products (except Organic NatureKnit™) compared to the negative control, which was mainly linked to an increase in members of the *Bacteroidaceae* family. Finally, the Actinobacteriota phylum was stimulated for all treatment conditions in the mucosal environment, associated with enhanced *Bifidobacteriaceae* abundance, with strongest effects observed following organic inulin supplementation reaching statistical and biological significance (*p* < 0.05 and FC > 4) for the *Bifidobacterium* genus.

#### 3.3.3 Alpha diversity

**Figure 5:**
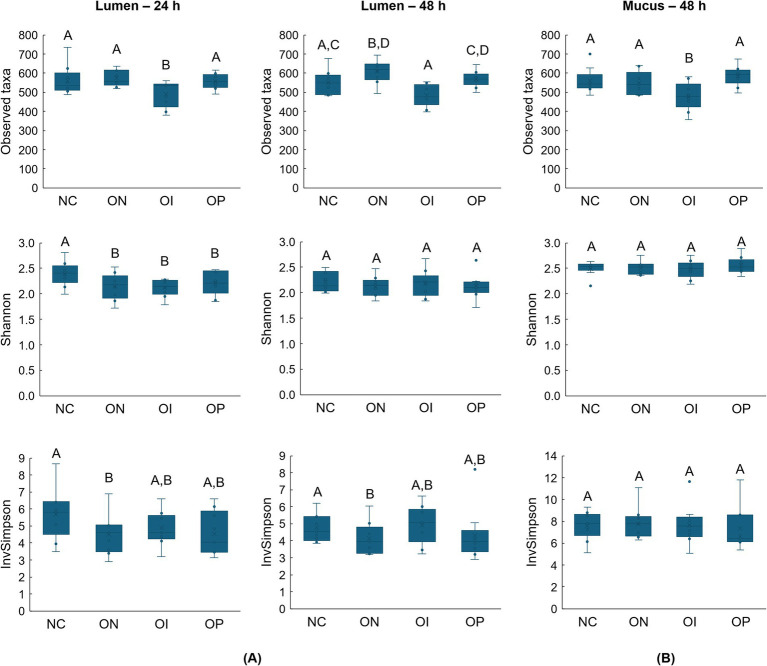
Effect of the test products on alpha diversity as calculated by the observed taxa, Shannon, and inverse Simpson indexes in the **(A)** lumen compartment (24 h and 48 h) and **(B)** mucosal compartment (48 h). Incubations included the negative control (colonic incubation blank medium), NatureKnit™ Organic (1.667 g fiber/L), organic inulin (1.667 g fiber/L), and organic psyllium (1.667 g fiber/L). Results are presented as mean ± standard deviation across donors (*n* = 9). Repeated measures on e-way ANOVA with *post hoc* Benjamini-Hochberg tests were used to compare the different test conditions. A *p*-value of <0.05 was considered statistically significant. When test conditions do not share a same letter, statistically significant differences between these test conditions were observed, while no significant differences were observed between test conditions that share a same letter. InvSimpson, inverse Simpson; NC, negative control; OI, organic inulin; ON, Organic NatureKnit™; OP, organic psyllium.

Alpha diversity results are shown in Figure 5. In the luminal compartment, the observed taxa index results were similar between the negative control and both NatureKnit™ Organic (*p* = 0.6565) and organic psyllium (*p* = 0.9171), but significantly decreased with organic inulin after 24 h of colonic incubation (*p* = 0.0339) (Figure 5A). Compared with the negative control, there was a significant increase in the observed taxa index with NatureKnit™ Organic (negative control, 549 observed taxa; Organic NatureKnit™, 619 observed taxa; *p* = 0.0495) at 48 h and no significant change with both organic inulin (475 observed taxa; *p* = 0.0567) and organic psyllium (569 observed taxa; *p* = 0.4285). For the Shannon diversity index, all test products had a significantly lower value compared with the negative control at 24 h (*p* = 0.0006, *p* = 0.0002 and *p* = 0.0234 for NatureKnit™ Organic, inulin and psyllium, respectively), and there were no significant differences among the conditions at 48 h (*p* = 0.0943, *p* = 0.7924 and *p* = 0.2268 for NatureKnit™ Organic, inulin and psyllium, respectively). The Inverse Simpson diversity index was significantly lower with NatureKnit™ Organic compared with the negative control at both 24 h and 48 h (*p* = 0.0128 and *p* = 0.0036, respectively), and similar between the negative control and organic inulin (*p* = 0.0564 and *p* = 0.7179, respectively) and organic psyllium (*p* = 0.0878 and *p* = 0.3241, respectively). In the mucus compartment (48 h), the observed taxa index was significantly lower following organic inulin supplementation versus the negative control (*p* = 0.0461); no significant differences were observed between NatureKnit™ Organic (*p* = 0.6046) or organic psyllium (*p* = 0.3191) compared with the negative control (Figure 5B). There were no significant differences among the test conditions with the Shannon or Inverse Simpson indices in the mucosal environment (*p* > 0.05 for all).

## 4 Discussion

Using a short-term single-stage colonic M-SHIME^®^ model, this study investigated the effects of supplementation with Organic NatureKnit™, a proprietary blend of diverse organic fruit and vegetable fibers rich in naturally occurring bound polyphenols, on microbial metabolism and community composition compared to supplementation with the organic version of the well-established prebiotic fibers inulin and psyllium. Reduced pH and increased gas pressure following supplementation with all three test products confirmed that they were fermented by the colon microbiota collected from each of the nine human donors. Each test product had a unique fermentation profile in terms of the kinetics of pH and gas pressure changes. Supplementation with each of the test products resulted in a significant increase in SCFA levels versus the negative control, which was most pronounced with NatureKnit™ Organic supplementation. The test products also induced a shift in the microbial community composition, with increases in the relative abundance of bacterial families that produce SCFAs. NatureKnit™ Organic supplementation resulted in a significant increase in total bacterial abundance and species richness versus the negative control at the end of the 48 h colonic incubation. This study both confirms previous findings that support NatureKnit™ as a prebiotic (35) and establishes that NatureKnit™ Organic acts in a similar manner as the non-organic product.

The slower, sustained decrease in pH that was observed with NatureKnit™ Organic indicates a more gradual fermentation for this product compared with the purified prebiotic fibers. Slower fermentation may impart additional health benefits compared with faster fermentation. For example, it is hypothesized that slower fermentation may benefit the gut by lowering the level of fiber fermentation in the proximal colon, and increasing the level in the distal colon (60) where the benefits of metabolic byproducts of fiber fermentation, such as SCFAs, are more pronounced (61). Considering that NatureKnit™ Organic includes both a diverse array of fibers (fruit and vegetable) and polyphenols and that the comparator products contain only purified fibers, we speculate that the presence of polyphenols may contribute to the slower fermentation observed with the NatureKnit™ Organic product. The gut microbial community can, in fact, break fiber-polyphenol bonds, allowing both the fibers and polyphenols to impact the colon. Several *in vitro* studies support synergistic effects between fibers and polyphenols (62, 63). However, these synergistic effects have yet to be explored in clinical trials. Our results indicate a potential additional benefit for natural fruit and vegetable fiber blends with bound polyphenols as compared to purified fibers. While this is supported by a previous *in vitro* study (35), additional studies are needed to more precisely confirm that NatureKnit™ is delivered to the distal colon, to understand the prebiotic potential of polyphenols, and to confirm the observed prebiotic effects in other *in vitro* simulation experiments.

Fermentation of high amounts of rapidly fermentable, purified fibers are associated with increases in flatulence and bloating in humans (64); however, the doses used in this study were below the amount that would be expected to cause discomfort. Change in gas pressure was greatest with NatureKnit™ Organic supplementation. Despite this, the overall gas pressure increases for all of the test products were below 100 kPa, which is considered an acceptable range.

While all three test products significantly increased SCFA levels compared with the negative control, NatureKnit™ Organic supplementation resulted in the greatest increase for each of the measured SCFAs (acetate, propionate, and butyrate). The observed increases in SCFA production are linked with increases in the luminal abundance of several SCFA-producing microbial families, including *Bifidobacteriaceae*, which contains members that produce acetate (65), and *Lachnospiraceae* and *Bacteroidaceae*, as well as mucosal stimulation of *Ruminococcaceae* abundance. *Lachnospiraceae* and *Ruminococcaceae* contain members that produce acetate, propionate, and/or butyrate (66, 67) and *Bacteroidaceae* contains members that produce acetate and/or propionate (68). Members of the *Lachnospiraceae* family, such as *Roseburia* and *Blautia*, are involved in controlling processes involved in gut inflammation through their production of butyrate (66, 69), making the expansion of this family of particular interest. SCFAs are quite beneficial to human health and butyrate, in particular, plays an important role in supporting intestinal epithelial cell health and maintaining an intact intestinal barrier (70, 71). Further, SCFAs are reported to have anti-obesity, anticancer, anti-diabetes, anti-inflammatory, immunoregulatory, hepatoprotective, cardioprotective, and neuroprotective activities (72). The increase in health-promoting SCFA levels with NatureKnit™ Organic supplementation, to an even greater extent than observed with the established organic prebiotics inulin and psyllium, supports a prebiotic role for Organic NatureKnit™.

NatureKnit™ Organic supplementation resulted in an increase in the number of different bacterial species in the gut microbiome (i.e., species richness), as evidenced by a significantly increased observed taxa value compared with the negative control at the end of the colonic incubation (48 h). Furthermore, significantly enhanced total bacterial abundance was observed following administration with NatureKnit™ Organic compared with the negative control, while the gold-standard inulin resulted in significantly lower total bacterial abundance at the end of the colonic incubation. Reduced gut microbiome diversity is associated with several diseases, negative health outcomes, and disorders (73, 74). For example, among older adults, reduced microbial diversity is associated with increased frailty (75). Patients with inflammatory bowel disease have reduced gut microbiome diversity, and diversity is increased when patients are in remission (76). Further, people who are obese, have inflammatory or rheumatic diseases, or have type II diabetes tend to have a less diverse gut microbiome compared with healthy controls (29, 77–81). Thus, an increase in gut microbiome diversity/species richness is generally considered beneficial for human health, further supporting the prebiotic potential of Organic NatureKnit™. Furthermore, similar to a previous study using conventional NatureKnit™, a consistent effect across donors could be observed compared to purified organic fibers. Although still a small sample size (*n* = 9), the consistency of the effect is likely a result of fiber and polyphenol diversity leading to greater bacterial interactions.

While these findings further support the prebiotic potential of NatureKnit™ and provide evidence that similar effects are seen with an organic formulation, future studies in humans are warranted to confirm whether such benefits are observed *in vivo*. Indeed, while *in vitro* simulations allow for in-depth mechanistic studies of product-specific effects on the gut microbial community activity and composition, findings from these studies do not translate directly to *in vivo* effects, for instance by lacking host interactions.

## 5 Conclusion

*In vitro* simulations of the human colon showed that NatureKnit™ Organic was fermented by the colon microbiota of nine individual healthy donors. A slower fermentation of NatureKnit™ Organic was suggested by the pH and gas pressure results. NatureKnit™ Organic demonstrated significant prebiotic effects, including stimulation of SCFA production, enhancements in microbial families that produce SCFAs, and an increase in bacterial total abundance as well as species richness. In line with previous findings with non-organic NatureKnit™ (35), the organic test product had effects that were equivalent to or more robust than two established prebiotics, inulin and psyllium. Overall, the *in vitro* study findings suggest that NatureKnit™ Organic may have a greater and gentler prebiotic effect than organic inulin or psyllium, which might predict, but does not confirm, reduced intestinal side effects in humans. These effects should therefore be further addressed in human clinical trials.

## Data Availability

The raw data supporting the conclusions of this article will be made available by the authors, without undue reservation.
