# Supplementary material for: Effects of NatureKnit™ organic, a blend of organic fruit and vegetable fibers rich in naturally occurring bound polyphenols, on the metabolic activity and community composition of the human gut microbiome using the M-SHIME® gastrointestinal model
Source: Front Nutr. 2026 Jan 16;12:1740906. doi: 10.3389/fnut.2025.1740906 (PMC12858181; doi:10.3389/fnut.2025.1740906)
Supplement: Supplementary file 1 [file Image_1.pdf]

Supplementary Material

1 Supplementary Figures

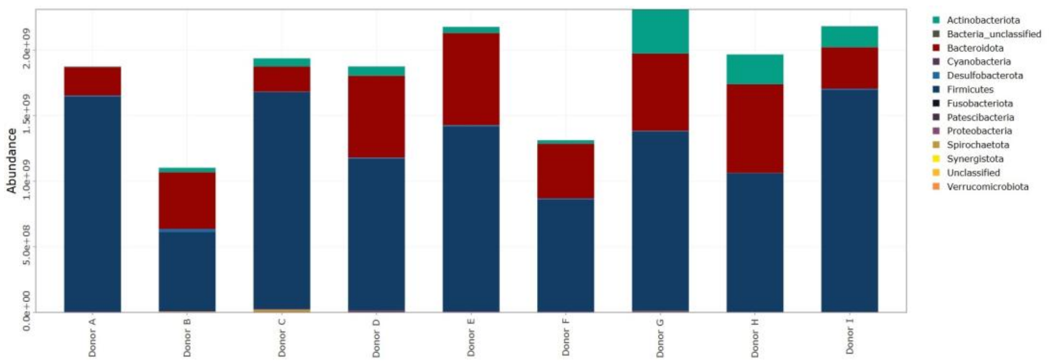

(A)

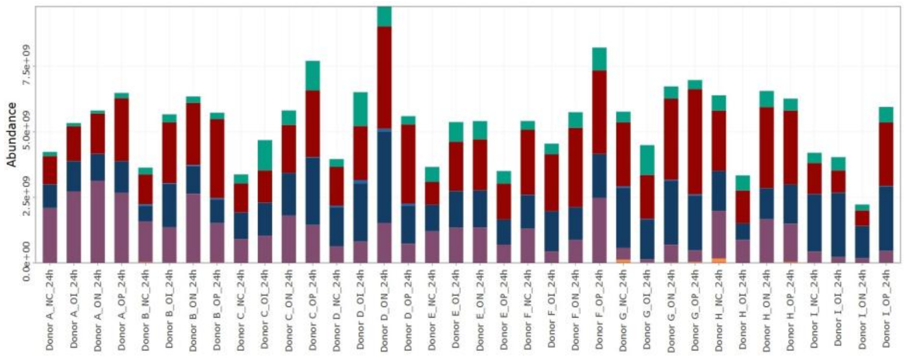

(B)

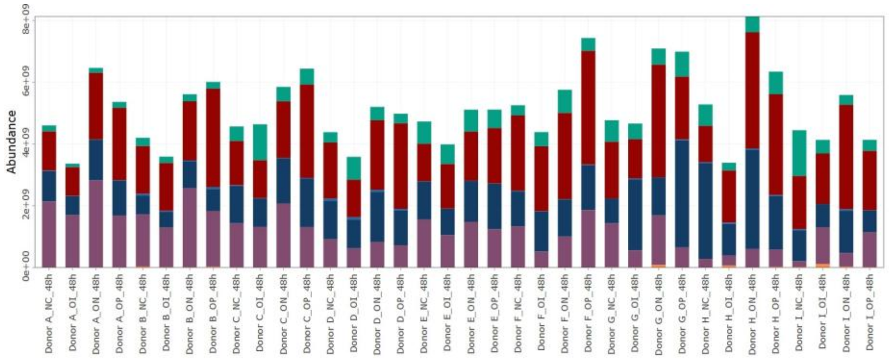

(C)

Supplementary Figure S1. Stacked bar plots showing absolute phyla abundances (cells/mL) in the lumen compartment at 0 h (a), 24 h (b), and 48 h (c). Incubations included the negative control (NC; colonic incubation blank medium), organic NatureKnit™ (ON; 1.667 g fiber/L), organic inulin (OI; 1.667 g fiber/L), and organic psyllium (OP; 1.667 g fiber/L). Results are presented for each individual donor (Donors A-I; n=1 per donor). Flow cytometry was used to determine the total number of bacterial cells in the luminal samples.

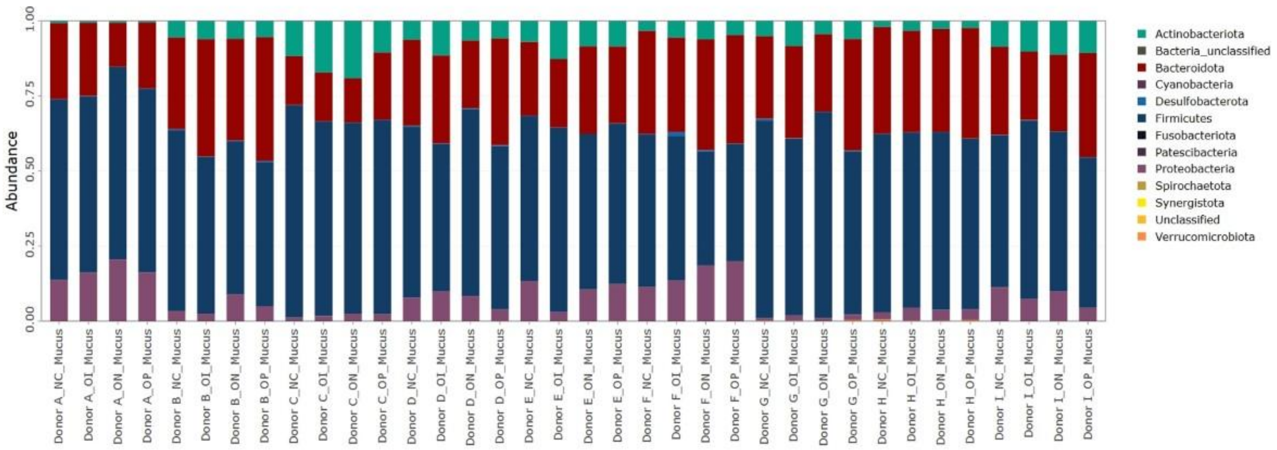

Supplementary Figure S2. Stacked bar plots showing relative phyla abundances in the mucosal compartment. Incubations included the negative control (NC; colonic incubation blank medium), organic NatureKnit™ (ON; 1.667 g fiber/L), organic inulin (OI; 1.667 g fiber/L), and organic psyllium (OP; 1.667 g fiber/L). Results are presented for each individual donor (Donors A-I; n=1 per donor).

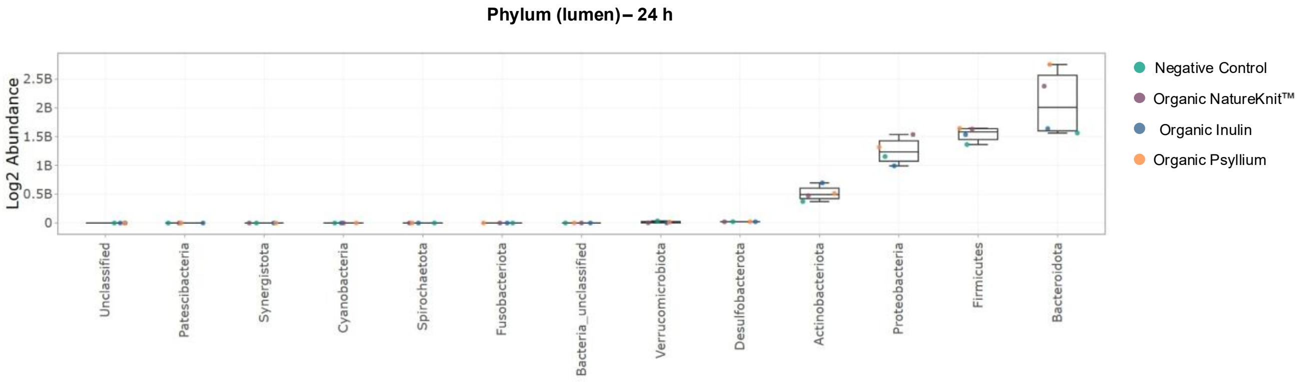

(A)

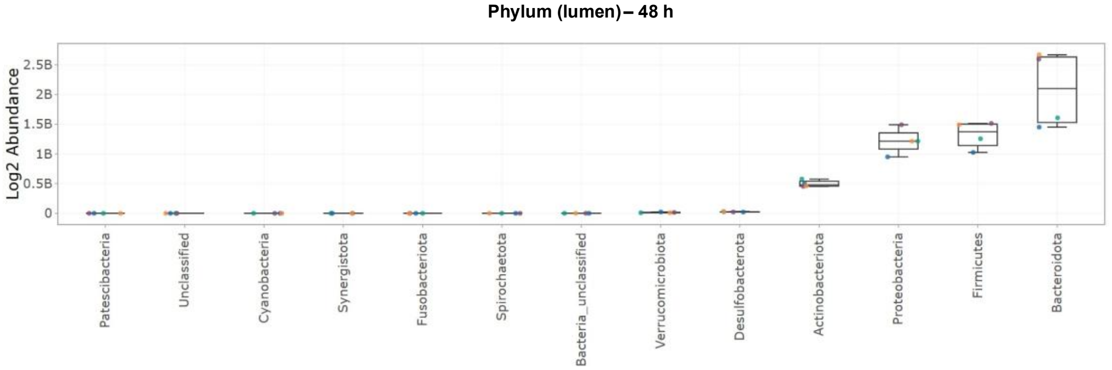

(B)

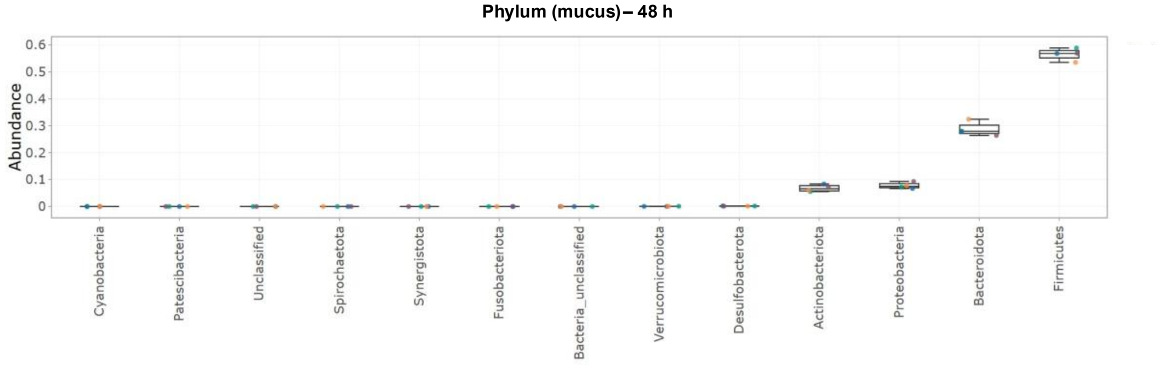

(C)

Supplementary Figure S3. Jitter plots showing (a) the absolute abundance (cells/mL) of the top 20 most abundant phyla in the lumen compartment after 24 h, (b) the absolute abundance (cells/mL) of the top 20 most abundant phyla in the lumen compartment after 48 h, and (c) the relative abundance (-) of the top 20 most abundant phyla in the mucosal compartment after 48 h. Incubations included the negative control (colonic incubation blank medium), NatureKnit™ Organic (1.667 g fiber/L), organic inulin (1.667 g fiber/L), and organic psyllium (1.667 g fiber/L). Each dot represents the average across donors (n=9).

2 Supplementary Tables

Supplementary Table S1. pH values (-) over time following test product administration in M-SHIME® short-term colonic incubations. Incubations included the negative control (colonic incubation blank medium), NatureKnit™ Organic (1.667 g fiber/L), organic inulin (1.667 g fiber/L), and organic psyllium (1.667 g fiber/L). Results are presented as average ± standard deviation (SD) across donors (n=9). M-SHIME® = Mucosal Simulator of the Human Intestinal Microbial Ecosystem; NC = negative control; OI = organic inulin; ON = Organic NatureKnit™; OP = organic psyllium.

		pH (mM)			
		0h	6h	24h	48h
Average	NC	6.57	6.45	6.47	6.50
	ON	6.57	6.25	6.20	6.19
	OI	6.57	6.21	6.22	6.25
	OP	6.57	6.42	6.34	6.34
SD	NC	0.01	0.04	0.02	0.02
	ON	0.01	0.06	0.03	0.04
	OI	0.01	0.12	0.02	0.03
	OP	0.01	0.04	0.02	0.03
